# Automatic identification of intestinal parasites in reptiles using microscopic stool images and convolutional neural networks

**DOI:** 10.1371/journal.pone.0271529

**Published:** 2022-08-04

**Authors:** Carla Parra, Felipe Grijalva, Bryan Núñez, Alejandra Núñez, Noel Pérez, Diego Benítez

**Affiliations:** 1 NuCom, Nuevas Comunicaciones Iberia S.A., Barcelona, Spain; 2 Faculty of Engineering and Applied Sciences (FICA), Telecommunications Engineering, Universidad de Las Américas (UDLA), Quito, Ecuador; 3 Departamento de Electrónica, Telecomunicaciones y Redes de Información (DETRI), Escuela Politécnica Nacional, Ladrón de Guevara, Quito, Ecuador; 4 Carrera de Medicina Veterinaria y Zootecnia, Universidad Técnica de Ambato, Ambato, Ecuador; 5 Colegio de Ciencias e Ingenierías “El Politécnico”, Universidad San Francisco de Quito USFQ, Quito, Ecuador; Universidad de Guadalajara, MEXICO

## Abstract

Captive environments trigger the propagation and multiplication of parasites among different reptile species, thus weakening their immune response and causing infections and diseases. Technological advances of convolutional neural networks have opened a new field for detecting and classifying diseases which have shown great potential to overcome the shortcomings of manual detection performed by experts. Therefore, we propose an approach to identify six captive reptiles parasitic agents (*Ophionyssus natricis*, *Blastocystis sp*, *Oxiurdo egg*, *Rhytidoides similis*, *Strongyloides*, *Taenia*) or the absence of such parasites from a microscope stool images dataset. Towards this end, we first use an image segmentation stage to detect the parasite within the image, which combines the Contrast Limited Adaptive Histogram Equalization (CLAHE) technique, the OTSU binarization method, and morphological operations. Then, we carry out a classification stage through MobileNet CNN under a transfer learning scheme. This method was validated on a stool image dataset containing 3616 images data samples and 26 videos from the six parasites mentioned above. The results obtained indicate that our transfer learning-based approach can learn a helpful representation from the dataset. We obtained an average accuracy of 94.26% across the seven classes (i.e., six parasitic agents and the absence of parasites), which statistically outperformed, at a 95% confidence level, a custom CNN trained from scratch.

## Introduction

Reptile parasitology research has not been fully explored in the scientific literature [1]. Parasites are one of the most common infectious agents and easily spread within wildlife management and care centers [2], which can cause injury or immune suppression in reptiles, increasing the mortality rate or leading to secondary diseases [3]. It has been shown that some types of parasites cause hepatitis in snakes [4]; others affect the behavior and physiology of the lizards, and even some parasites can cause chronic enteritis with edema and hemorrhagic intestinal mucosa [4]. Also, mites in reptiles cause weakness due to blood loss, pneumonia, and even septicemia [5]. The need to control parasitism in captive species is important because they can be a source of transmission of zoonotic diseases, which in turn may be transmitted from animal to human [6]. Manual parasite classification methods involve analyzing stool microscope images by experts. However, this task requires significant effort and time since, in a center where reptiles are sheltered, they may have a very high parasite load of different types, so that it could affect their health. Automatic classification tools for parasitic animal agents can help researchers or keepers to operate much faster and efficiently. Consequently, it is possible to perform faster diagnoses and prepare the adequate deworming protocols to prevent diseases in captive reptiles.

In this context, Convolutional Neural Networks (CNNs) have proven to be a valuable tool to find and classify patterns in images easier than the specialists, which invest long identification times prone to error. CNNs have been studied for several years, achieving good results in image classification tasks, and by using similar models, it is possible to generalize algorithms to solve different types of problems [7]. For example, these machine learning tools have recently been tested in many fields including medicine and biology [8, 9]. There are several factors [10] that influence the effectiveness of CNNs (e.g., architecture, data processing, segmentation). Moreover, the complexity involved in training a CNN from scratch is in general not feasible when the data are insufficient. Therefore, this has led to the use of supporting methods such as data augmentation and transfer learning [11]. The latter uses a previously trained CNN with a base task, and then fine-tune it on a target task [12].

In light of this, this paper proposes a new approach for identifying parasitic agents from microscopic stool images or the absence of parasites that affect reptiles in captivity, through segmentation algorithms, data augmentation strategies, and CNNs under a transfer learning scheme.

### Related work

This section describes several previous studies on similar domains as our approach employing image processing and machine learning techniques to classify different species, including intestinal parasites from fecal samples, as in our case. For example, in [13] an in‑clinic canine and feline fecal parasite detection system integrated with a deep learning-based algorithm was used to locate, classify, and identify parasite eggs (i.e. *Ancylostoma*, *Toxocara*, *Trichuris* and taeniid eggs) found on fecal microscopic slides. An object detection network based on Single Shot MultiBox Detector (SSD) with Inception v2 deep learning backbone was used for localization and classification. Classification accuracy of 93.8% was achieved across the four targeted parasites in a data set of 100 fecal samples containing a minimum of 10 fecal samples for each targeted parasite. Similarly, in [14], a low-cost, automated parasite diagnostic system using fecal samples of sheep via a portable robotic microscope and a CNN based on the U-Net structure is presented. The system was trained with egg parasite morphologies of ascarid, *Trichuris spp.*, strongyle, and *Coccidia*, achieving an accuracy of 92% to 96%.

Previous works [15] also investigated human intestinal parasites classification using deep belief networks over three datasets composed of Helminth eggs, Helminth larvae, and protozoan cysts, achieving about 94% of balanced accuracy score, even with considering unbalanced classes and also fecal impurities. In [16], a ResNet152 residual network was used for the detection and identification of visible components in fecal microscopic images, including Hookworm eggs, Ascarid eggs, and Whipworm eggs, amongst other fecal components, reporting mean average precision (mAP) and an average recall (AR) of 89.95% and 93.88%, 96.90% and 91.21%, and 88.61% and 94.37%, respectively.

Similarly, in [17], deep convolutional neural networks were used for the diagnosis of Malaria in thick blood smears, tuberculosis in sputum samples, and intestinal parasite (hookworm) eggs in stool samples, reporting area under the curve (AUC) up to 100% for Malaria and 99% for tuberculosis and hookworm and average classification precisions of 97%, 93%, and 93%, respectively. While in [18], the authors present a method for the detection and binary classification of cells infected by the malaria parasite in blood images. They propose a segmentation stage based on morphological top-hat operators [19], and the classification stage uses different sets of texture and shape features that feed a neural network. [9] presents a survey of deep learning applications for medical image processing, including histological and microscopical elements detection, such as parasites detection in stained blood smear samples.

Furthermore, image processing and deep learning-based algorithms have also been used to identify and classify tiny insects. For example, in [20], a model for the detection of adult whitefly (*Bemisia tabaci*) and thrips (*Frankliniella occidentalis*) in the greenhouses was proposed. An image acquisition system using adhesive traps allowed the collection of the database. Segmentation was performed using the OTSU algorithm and other digital image processing methods. Finally, the classification was carried out with the help of a feed-forward neural network. Similar work is addressed by [21], where several morphological features related to the size and color of the specimens were extracted and analyzed to classify them.

Moreover, in [22], a real-time remote insect trap monitoring system employing IoT and a method for classifying insects based on a Faster region-based CNN (R-CNN) and ResNet 50, applying transfer learning was proposed. The results show that the system could automatically identify insects with 94% accuracy.

Finally, in [23] an approach to classify protozoa and metazoa organisms in wastewater treatment plants was proposed. Specifically, they compare discriminant analysis, neural networks and decision trees. They found that the discriminant analysis and the neural network performances were quite similar, while the decision tree technique was less efficient.

Despite these efforts, as far as we know, this is the first attempt to build a machine learning model to specifically classify reptilian parasites using convolutional neural networks from microscopic stool images. For this, as in previous works, we will use traditional digital image processing techniques such as binarization and segmentation [18, 20, 23] in a pre-processing stage to prepare image samples for our classifier. Then, we are going to use a CNN-based classifier under a transfer learning scheme as in [22]. The contributions of this paper are related to (a) the unprecedented study of an end-to-end machine learning model based on image segmentation and CNNs for learning expressive features from microscopic stool images to discriminate reptilian parasites; (b) the comparison of a transfer learning scheme against a custom CNN training from scratch, and (c) the introduction of a new public dataset containing microscopic stool images collected and annotated by experts.

## Materials and methods

In this section we first describe in detail the dataset from the stool sample collection to the labeling process by an expert veterinarian. Next, we explain the stages of the proposed method, and finally we introduce the experimental setup to evaluate our methodology.

### Dataset

**Fig 1:**
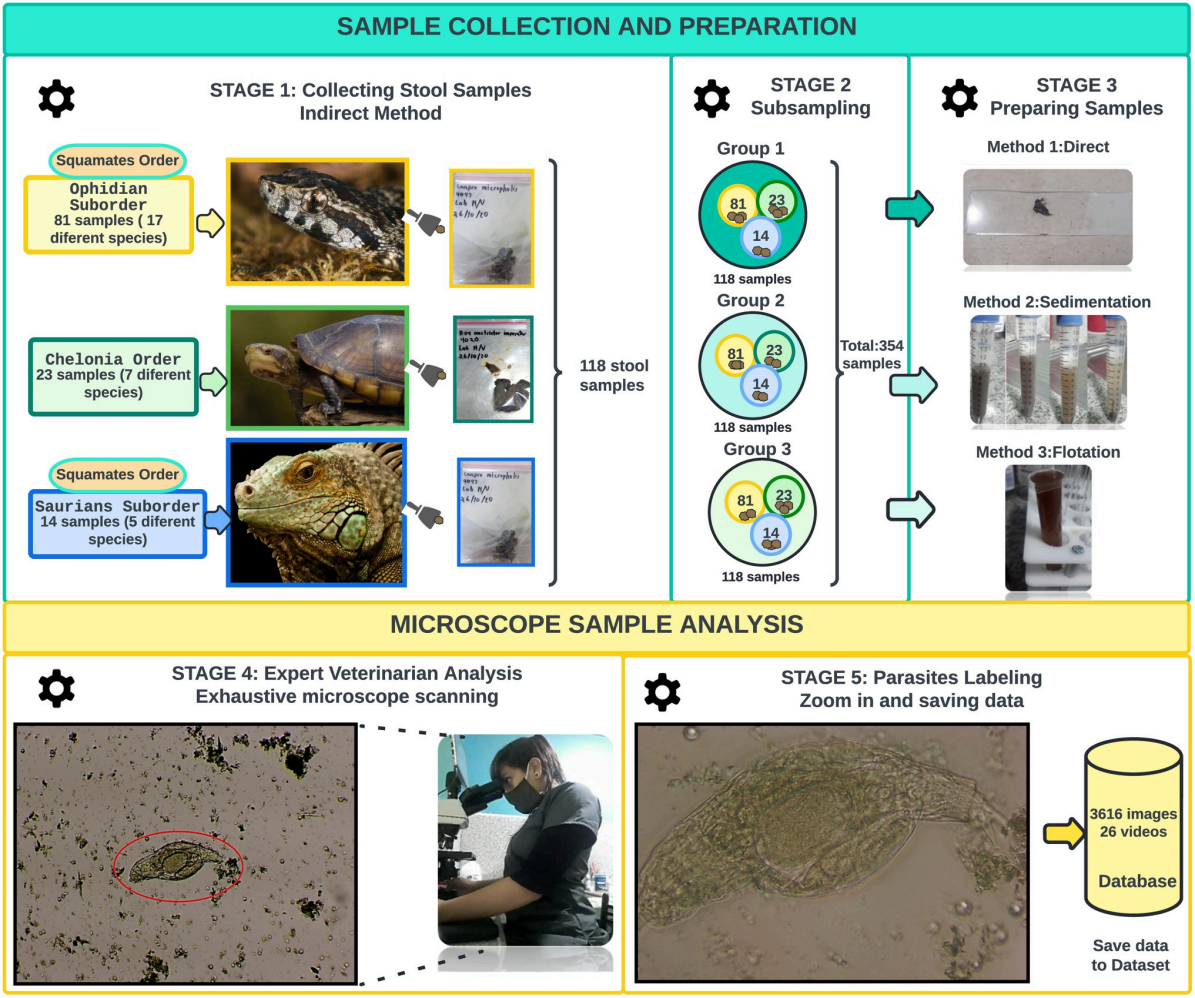
Dataset collection procedure.

The dataset collection is a five-stage procedure from stool sample collection and preparation to microscope sample analysis as shown in Fig 1. The dataset contains images of coproparasitic samples of two orders of reptiles: *Chelonians* (e.g., aquatic, semi-aquatic and terrestrial turtles) and *Squamates* (*Ophidians* such as venomous and non-venomous snakes, and *Saurians* such as lizards, geckos and iguanas) that live in captivity in the Vivarium in Quito (Ecuador) https://vivarium.org.ec, which houses approximately 350 animals of different species of amphibians and reptiles.

During the first stage, the collection of stool samples was carried out by the indirect method, i.e., from the samples of stool deposited in the areas where each animal is located. Since the indirect method employed in this study does not involve the collection of samples directly from the animal, ethical approval was not required. The samples were collected with a tongue depressor for each sample to avoid contamination, and then they were stored within vacuum-sealed Ziploc bags. These bags were labeled and put into an airtight cooler container, and then stored at a temperature between 1°C to 6°C. In total, 118 stool samples were collected, out of which 81 samples were collected from 17 different species of the suborder *Ophidians*, 23 samples belong to 7 different species of the order *Chelonians*, and 14 samples correspond to 5 different species of the suborder *Saurians*.

In the second stage, a sub-sampling procedure was performed, i.e., each sample was divided into three new independent samples. So, we ended up with three different groups of 118 samples per group (i.e., a total of 354 stool samples) with the same order and suborder distribution as the 118 original stool samples from stage 1.

In stage three, the stool samples have been prepared before being analyzed under the microscope. For this purpose, three widely used methods have been used to diagnose parasitic infections. Since some method makes it possible to identify some parasites better than others, each of the three groups of samples was analyzed by one of the three methods. Concretely, samples from group 1 were processed by the direct method, where the fresh stool samples were directly placed under the microscope to look for mostly mobile parasite forms. Samples from the second group were prepared using the flotation technique method, which causes the parasite forms to float to the surface due to their lower density compared to the density of the solution in which they have been immersed. Finally, samples from group 3 were prepared using the sedimentation technique, in which parasites naturally settled by gravity in a medium of lower density.

In stage four, an expert veterinarian carried out an exhaustive scanning along with each sample to find known parasitic forms using a digital tactile microscope (Better Scientific Led Q190A-LCD, magnification of X10, X40, and X100).

Finally, in stage 5, when a parasite was found according to the criteria of an expert veterinarian, the specialist zoomed in to better capture the parasite under the microscope and saved an image or video from it with its corresponding label.

**Fig 2:**
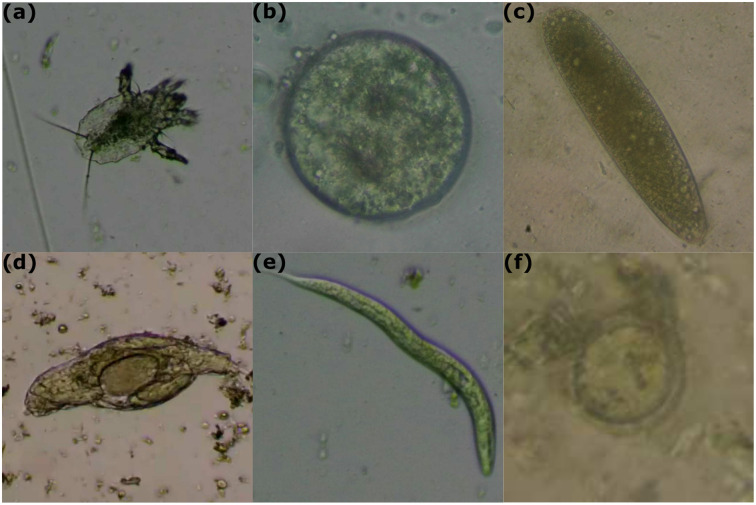
Examples of each parasite. (a) Acaro (*Ophionyssus natricis*); (b) *Blastocystis sp*; (c) *Oxiurdo egg*; (d) *Rhytidoides similis*; (e) *Strongyloides*; (f) *Taenia*.

The aforementioned sample collection, preparation, and labeling processes were performed by an expert veterinarian (author A. Núñez) specifically for this study. The specialist manually labeled 3616 images and 26 videos containing 4849 frames from six parasites (see Fig 2 for examples of each parasite) according to the distribution shown in Table 1. Video frames that did not contain a parasite were removed. It is worth noting that each image or frame contains only one parasite in our dataset.

**Table 1:** Parasites database distribution.

Parasites (labels)	Total images	Videos
Mite—*Ophionyssus natricis* (ophi)	245	2 (1236 frames)
*Blastocystis sp* (blas)	950	0
*Oxiurdo egg* (oxi)	345	6 (888 frames)
*Rhytidoides similis* (rhyti)	1072	6 (1048 frames)
*Strongyloides* (strong)	648	11 (764 frames)
*Taenia* (tae)	356	1 (913 frames)
**Total**	3616	4849

### Proposed method

**Fig 3:**
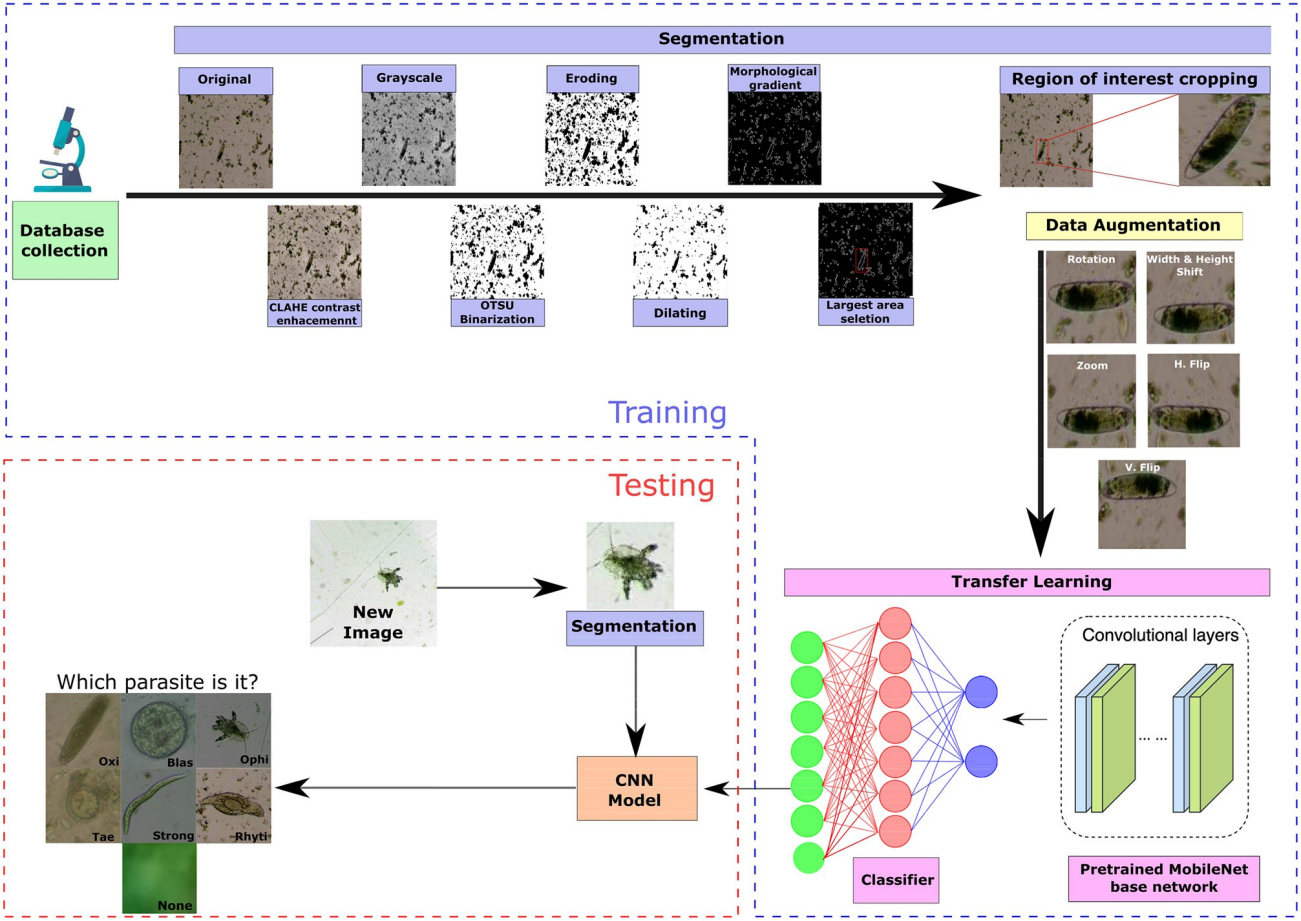
Block diagram of the proposed approach.

Our proposed approach is composed of several stages as shown in Fig 3. The first stage involves collecting images from reptile feces. A veterinarian specialist labeled the images according to the parasite present in them. These images must pass through a segmentation process to reduce background noise to appreciate the parasite as clearly as possible. Also, they must go through a process of data augmentation to deal with the unbalanced classes. The pre-trained MobileNet architecture is used to train a new model on our images in the transfer learning stage. Finally, the trained model predicts the type of parasite for previously unseen reptile feces images or the absence of parasites (i.e., hereinafter called *None* class). Next, we describe in detail each of these stages.

#### Region segmentation

**Fig 4:**
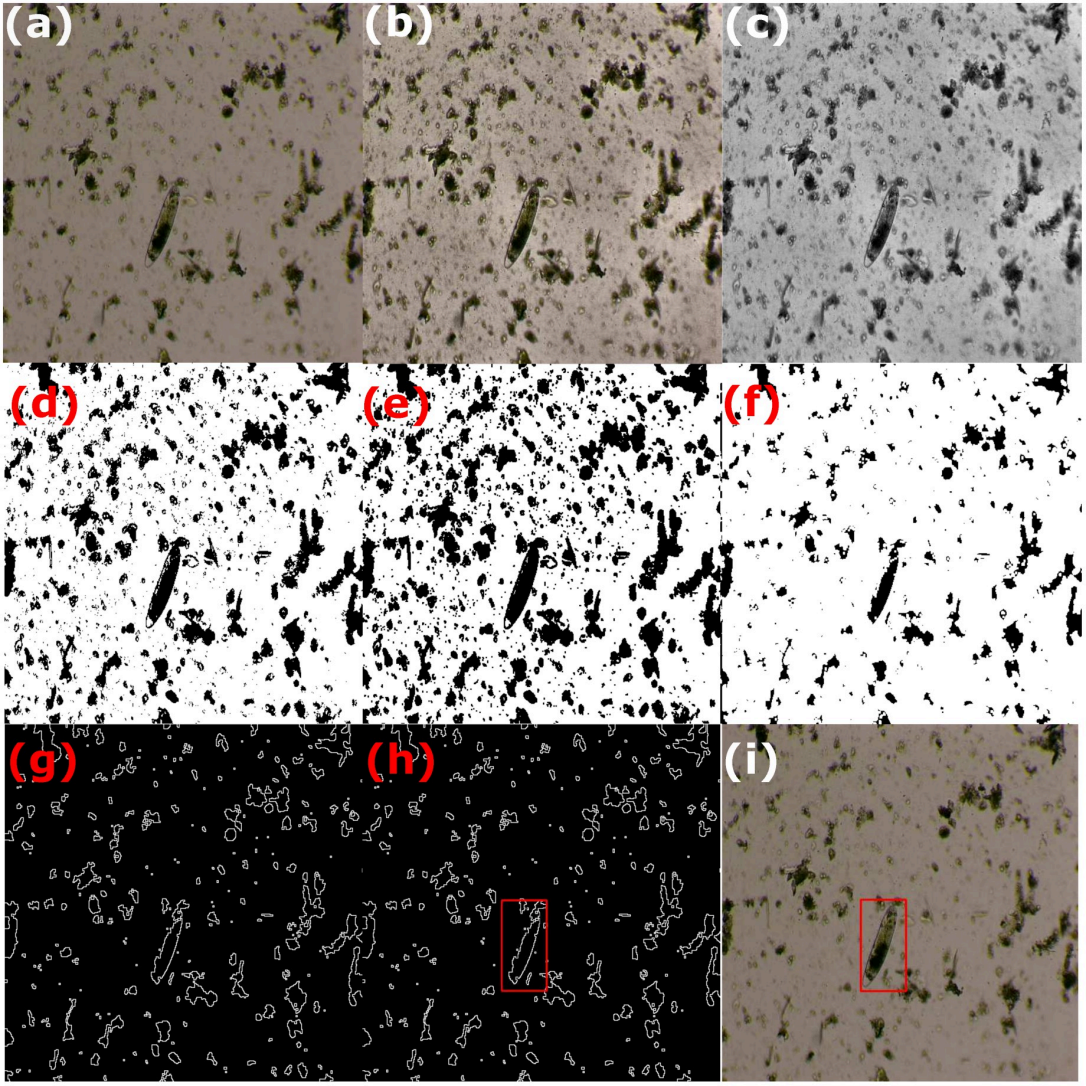
Parasite detection using image processing techniques: (a) original image; (b) image contrast enhancement using CLAHE; (c) color to grayscale image conversion; (d) image binarization using the OTSU method; (e) erode-based morphological operation; (f) dilate-based morphological operation; (g) morphological gradient operation; (h) the largest areas are strong candidate regions to contain the region of interest (the image depicts only the largest one), and (i) plotting the region of interest in the original image.

Segmentation is a digital image procedure that extracts the region of interest from the original image [24]. The segmentation stage was crucial to achieving good performance in the network training stage since most of the images in the database are very noisy (e.g., as shown in Fig 4a) since the images were taken from animal feces. With this aim, the images were processed by the following steps as depicted in Fig 4.

**Fig 5:**
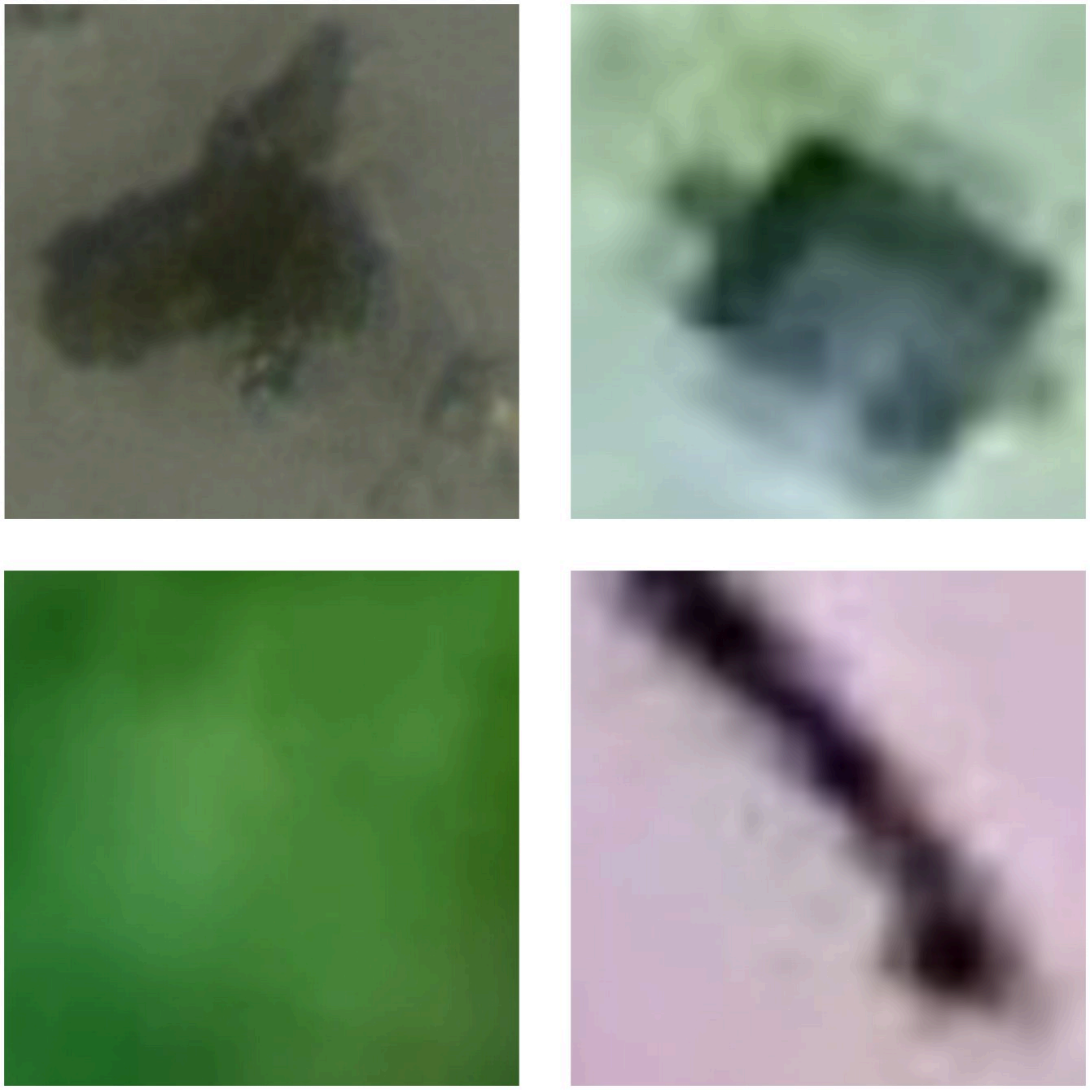
Some examples of samples from the None class. They normally correspond to large pieces of debris or garbage.

- CLAHE (Contrast Limited Adaptive Histogram Equalization) is a digital image processing technique that improves the image contrast without increasing noise. It selects different sections of the image to redistribute their pixel brightness values. As a result, the image contrast is improved while preserving the contours of the objects [25], as shown in Fig 4b.
- After converting the RGB (red, green, blue) color image to grayscale, the image binarization through the Otsu’s method [26] is applied, which determines the most appropriate conversion threshold by minimizing the intra-class variance between two assumed pixel classes (usually, black and white), as shown in Fig 4d.
- Morphological operations [27] (Fig 4e and 4f) are performed by eroding and then dilating the image pixels in order to decrease noise in the image through the kernel
  $$\begin{array}{c} B=\lceil \begin{array}{ccc} 1 & 1 & 1\\ 1 & 1 & 1\\ 1 & 1 & 1 \end{array}\rceil \end{array}$$
  Observe that erosion tends to remove small objects due to debris or garbage so that only substantive objects remain, whereas dilation makes objects such as the parasite more visible.
  The morphological gradient (Fig 4g) of an image *I* is then obtained by calculating the difference between the dilation (⊕) and erode (⊖) operations from the previous step using the kernel *B*, according to the following equation
  $$\begin{array}{c} \text{G}=\text{(I}⊕\text{B)}-\text{(I}⊖\text{B)}\;\forall \text{I}\in R^{2} \end{array}$$
  In the resulting image *G*, the contours of the most significant objects are emphasized [28].
- Finally, the areas of possible objects are computed after the morphological gradient operation application. We pick the top eight largest areas to feed the neural network classifier because it is very likely that one of them contains the target object. For instance, Fig 4h and 4i. depicts how one of the largest areas might contain the parasite. Since, at test time, it may occur that the majority or even all of the eight largest areas do not enclose any parasite due to some sufficiently large debris or garbage within the image, the neural network includes a no parasite class. Thus, we have added 1744 no parasite examples to the dataset from the largest areas detected by our region segmenter with no parasite within them. They normally correspond to large pieces of debris or garbage (see Fig 5) as verified by our expert veterinarian.

Although the segmentation stage ensures that our system automatically finds a set of candidate regions containing a parasite during the testing time, in order to train the neural network, our expert veterinarian manually verified that the segmented images and video frames from the database training data were segmented correctly. If a training example was wrongly segmented, our expert veterinarian manually segmented that image. Hence, we guarantee that the ground truth labels are entirely correct during training while avoiding biases in our classification model.

#### Data augmentation

The objective of data augmentation techniques is to generate new image samples by transforming the original one. Usually, these transformations are affine transformations, i.e., projective transformations that do not move the objects of the image [29]. Thus, they preserve the collinearity of the objects features in the space under analysis. At present, data augmentation is a practical solution to train deep network models which demand a vast amount of image samples, avoiding model overfitting. We used the following augmentation strategies:

- Rotation range: It is the degree range for random rotations. We used a range between -180 to +180 degrees.
- Width shift range: It randomly shifts an image to the left or right by a proportional percentage of the image width. This value was set to 0.2, i.e., 20% of the image width.
- Height shift range: Similar to the previous transformation, but the shift is up or down. This value was set to 0.2.
- Zoom range: It allows for varying the zoom of an image randomly. This value was set to 0.2, i.e., the zoom range lies between 80% to 120%.
- Horizontal flip: It randomly allows an image to be flipped horizontally.
- Vertical flip: It randomly allows an image to be flipped vertically.

Through this process, we augmented the dataset instances so that each class has roughly 1500 images, including original images, augmented images, and video frames to increase the opportunity for a better model’s performance. Since the videos contain parasites in movement, which are visually similar to resulting images from data augmentation procedures, we avoid using data augmentation strategies in the video samples.

Finally, for the rhyti class, the data augmentation algorithms were not used since the instances from this class in conjunction with video frames already reached more than 2000 images. For a similar reason, we did not augment the None class. After performing these procedures, a total of 11843 images were obtained with the distribution shown in Table 2.

**Table 2:** Total number of images in the parasites database after data augmentation.

Parasites Class	Original Images	Augmented Images	Video Frames	Total Images
ophi	245	118	1236	1599
blas	950	550	0	1500
oxi	345	402	888	1635
rhyti	1072	0	1048	2120
strong	648	262	764	1674
tae	356	302	913	1571
None	1744	0	0	1744
Total	5360	1634	4849	**11843**

#### Region classification using transfer learning with the MobileNet CNN

Commonly, machine learning is constrained to use the same input feature space for training and testing developed models. Otherwise, the predictive performance of the classifier is degraded due to any difference in data (train and test) distributions [8]. In some problems, such as the reptile intestinal parasite classification, collecting training and testing data that matches the feature space while conserving the same data distribution can be tough (the target object is microscopic) and expensive (laboratory experts and equipment). Thus, creating a high-performance classifier (learner) for a specific target domain trained from a related source domain is a beneficial transferred learning solution.

**Fig 6:**
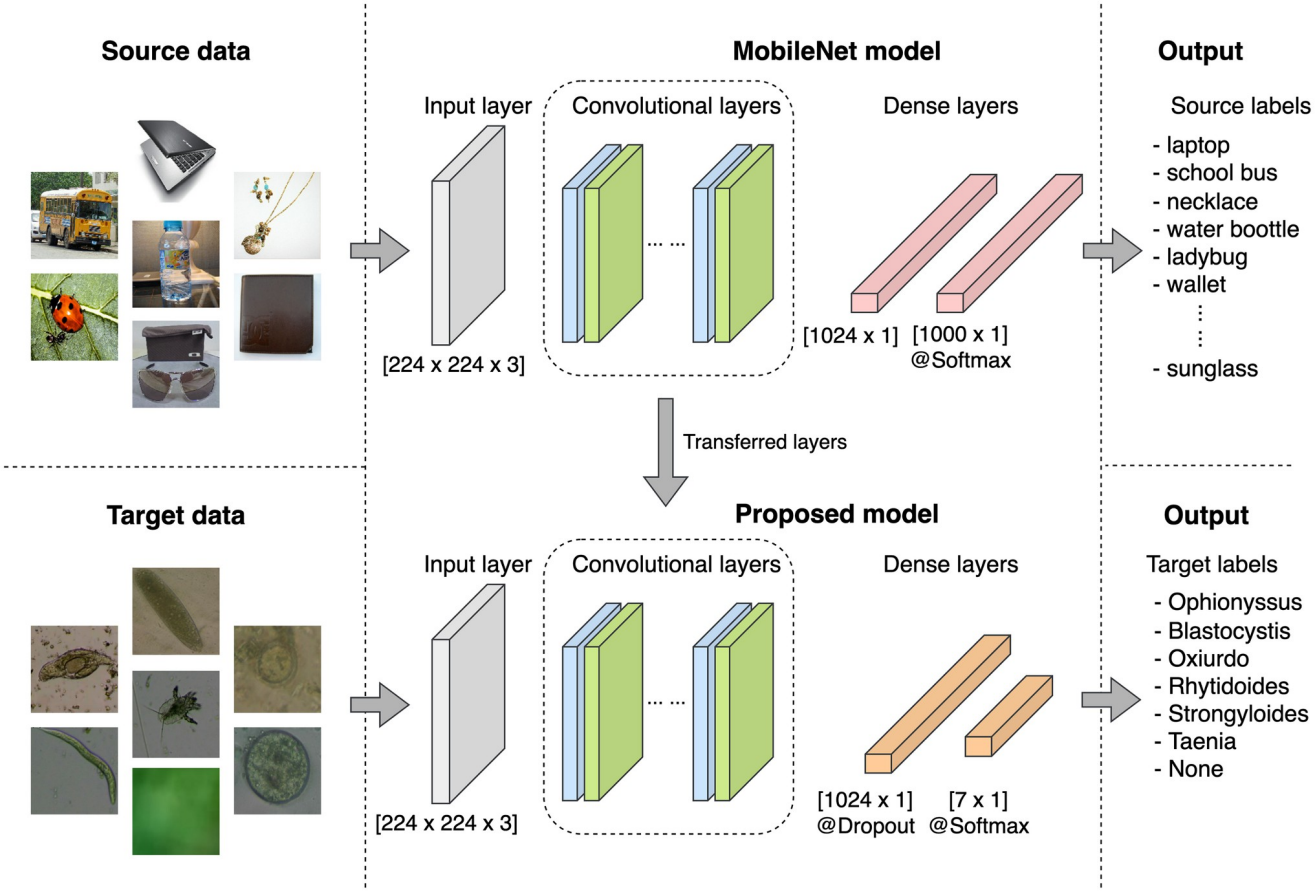
Workflow of the transferred learning solution.

In this way, as the region classification is a specific subtask of the overall object classification, we considered the ImageNet database [30] as the source domain and the reptile intestinal parasite data as the target domain, as shown in Fig 6. Also, we used the MobileNet [31] architecture as the classifier, which is a CNN model trained with the ImageNet database, containing about 14 million images. This model can provide classification performances comparable to other more robust artificial neural networks such as ResNet [32] or VGG16 [33]. However, it consumes less computational resources thanks to the depthwise separable convolution blocks (DWSCB). These blocks reduce the calculation time (approximately 8 to 9 times) concerning a standard convolution operation [31], making it suitable for limited resource devices [31].

MobileNet was designed to classify 1000 classes of objects. Thus, the later step of the model belonging to the classifier is irrelevant to our multiclass classification problem. In opposite, we transfer all convolutional layers (depthwise separable and standard blocks) with their settings and weights to a similar model that modifies the classifier to handle seven classes (see Table 2). Six of them belong to parasites. One class called *None* represents the open set (non-existence of parasites). The main advancement of the new classifier is the inclusion of a dropout layer with a rate value of 25% to improve the generalization error and to avoid overfitting [34], as shown in Fig 6. Transferring only the object feature extractor part of the pre-trained MobileNet model allows us to avoid long training time and to fine-tune the new weights of the proposed model. Moreover, the use of a transfer learning solution helps us separately transform both domains (source and target) into a common latent feature space that unifies the input space of the developed classifier.

### Experimental setup

This section describes the experimentation methodology employed to assess the proposed approach, such as data partition, data augmentation, neural network configuration, comparison with a custom CNN model and the evaluation metrics.

#### Data partition

We have used the experimental dataset described in Table 2, which is composed of 11843 instances including original images, augmented images and video frames. We have applied a stratified five-fold cross-validation method [35] (*k* = 5) on the experimental dataset to secure disjoint sets (training and test) and to ensure that the seven classes have proportional representation on each fold.

#### Neural network configuration

Since we use transfer learning with the MobileNet that was previously trained with different images (i.e., from the Image Net dataset), a parameter tuning process was performed for all layers in the network. Other hyperparameters and settings were stated as:

- Optimization Algorithm: The Adam method was used as it works better than the common stochastic gradient descent. It adapts the learning rate while training for different parameters from first and second-moment estimates of the gradients [36].
- Learning rate: This hyperparameter allows the weights to be updated for each epoch during the training of a neural network. We used a small learning rate as suggested by [37] with an initial value of 0.001. This hyperparameter was reduced by a factor of 0.5 whether the network had not improved its accuracy in 2 epochs.
- Loss function: Since this classification problem is multiclass, the categorical cross-entropy was chosen as the loss function since it leads to faster training as well as improved generalization for classification tasks [38].

#### Baseline CNN model

**Fig 7:**
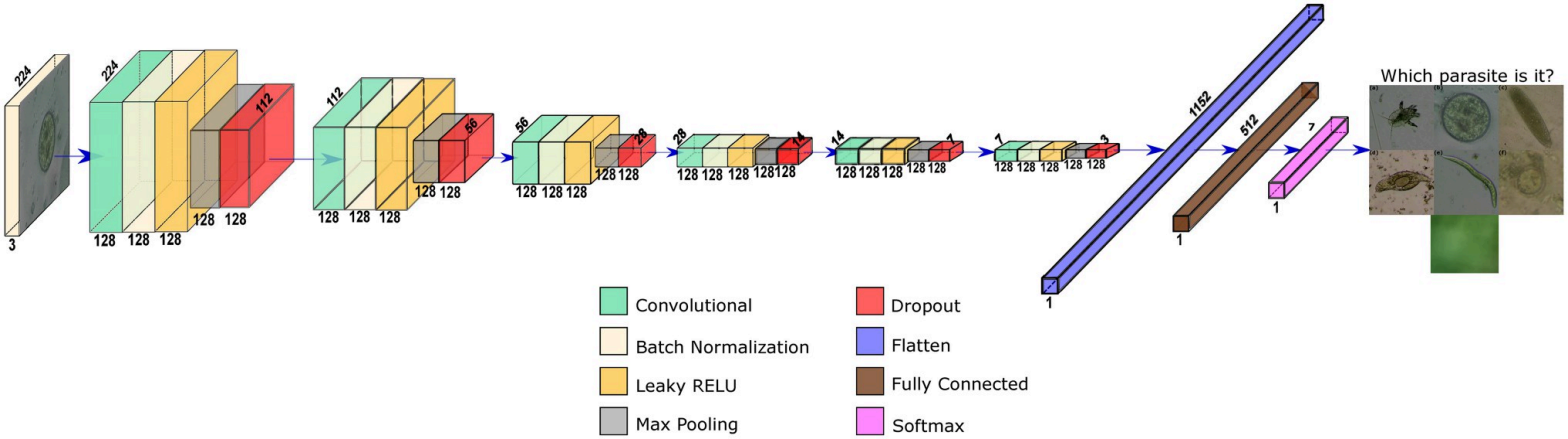
Custom CNN for *C* = 6 blocks and *F* = 128 filters.

We trained a custom CNN, built from scratch to compare against the transfer learning scheme with MobileNet. This network receives 224x224 images as input and has seven outputs classes, similar to the MobileNet network. The custom network has *C* convolutional blocks as shown in Fig 7. Each block is composed of the following layers:

- Convolutional layer with *F* filters of size 3 × 3. We set the strides values to 1x1 and zero padding such that the output has the same dimensions as the input.
- Batch Normalization Layer (BN) layer that acts as a regularizer to avoid overfitting and primarily enables training with higher learning rates, which is the cause of faster convergence and better generalization [39].
- Leaky ReLU Layer with a slope of 0.3 has a faster calculation speed and convergence rate, unlike other activation functions such as the sigmoid, thanks to its linearity. Also, this layer was introduced to avoid the vanishing gradient problem since this layer does not cause saturation for negative and positive inputs [40].
- Max Pooling layer allows reducing the dimensionality of the feature maps by summarizing the most active presence of a feature [41].
- Dropout Layer with a drop rate of 0.25 that helps to prevent overfitting [34].

After the convolutional blocks, a flatten layer and one dense layer of 512 nodes with Leaky ReLU activation were added. Finally, the output layer is a seven-node dense layer with softmax activation to discriminate each of the six types of parasites or the absence of parasites (i.e. the None class).

We varied *C* from 3 to 6 convolutional blocks by keeping constant *F* = 128 filters to explore how depth affects the network performance. Then, we kept constant *C* = 6 convolutional blocks and varied *F* for 32, 64, and 128 filters to analyze the impact of filter size on the network.

#### Evaluation metrics

We used the Area Under the Curve (AUC) metric obtained from the ROC (Receiver Operating Characteristic) curve on the same five-fold cross-validation partitions for both the custom CNN and the transfer learning architectures. The ROC curve was obtained by plotting the sensitivity or true positive rate (TPR) on the y-axis against the specificity or false positive rate (FPR) on the x-axis for different threshold decision values varying from 0 to 1.

Moreover, in order to guarantee a fair and statistically reliable comparison, we repeated four times (with different random seeds) the five-fold cross-validation partition scheme, giving a total of 20 runs for each neural network architecture. Since we deal with a multiclass problem, we used the micro-average AUC to compare the transfer learning scheme against the custom CNN. Finally, we calculated the confusion matrix from a five-fold cross-validation run and the overall accuracy of all runs.

## Results and discussion

Before presenting the results with our transfer learning scheme, we present the results of the hyperparameter optimization of the number of convolutional blocks *C* and the number of filters *F* from the custom CNN. In Table 3, we show the average accuracy for different values of *C* and *F* across the 20 runs with different data partitions. We also show the number of trainable parameters for each experiment. Observe that as the number of filters increases, the average accuracy also increases. To select the best custom CNN model, we selected the one with the best average accuracy. In case there are statistically similar results according to the t-test at 95% accuracy, we chose the one with the lowest number of trainable parameters. According to this strategy, the best model is achieved with 128 filters and 6 convolutional blocks, which hereinafter will be referred as the optimized custom CNN.

**Table 3:** Average accuracy and standard deviation (std) with their respective number of trainable parameters and p-values for different values of C and F across 20 runs with different random data partitions. P-values are calculated using 128 filters and 6 convolution blocks as the pivot.

Filters *F*	Conv. blocks *C*	Accuracy (std)	Trainable param	P-value
128	3	80.03 (4.72)	51,683,847	0.00996
128	4	81.97 (3.74)	13,296,519	0.0981
128	5	80.62 (2.37)	3,810,567	0.00155
128	6	83.96 (1.63)	1,333,376	–
64	6	81.90 (2.20)	486,215	0.034
32	6	74.85 (2.40)	199,079	7.3e-6

**Fig 8:**
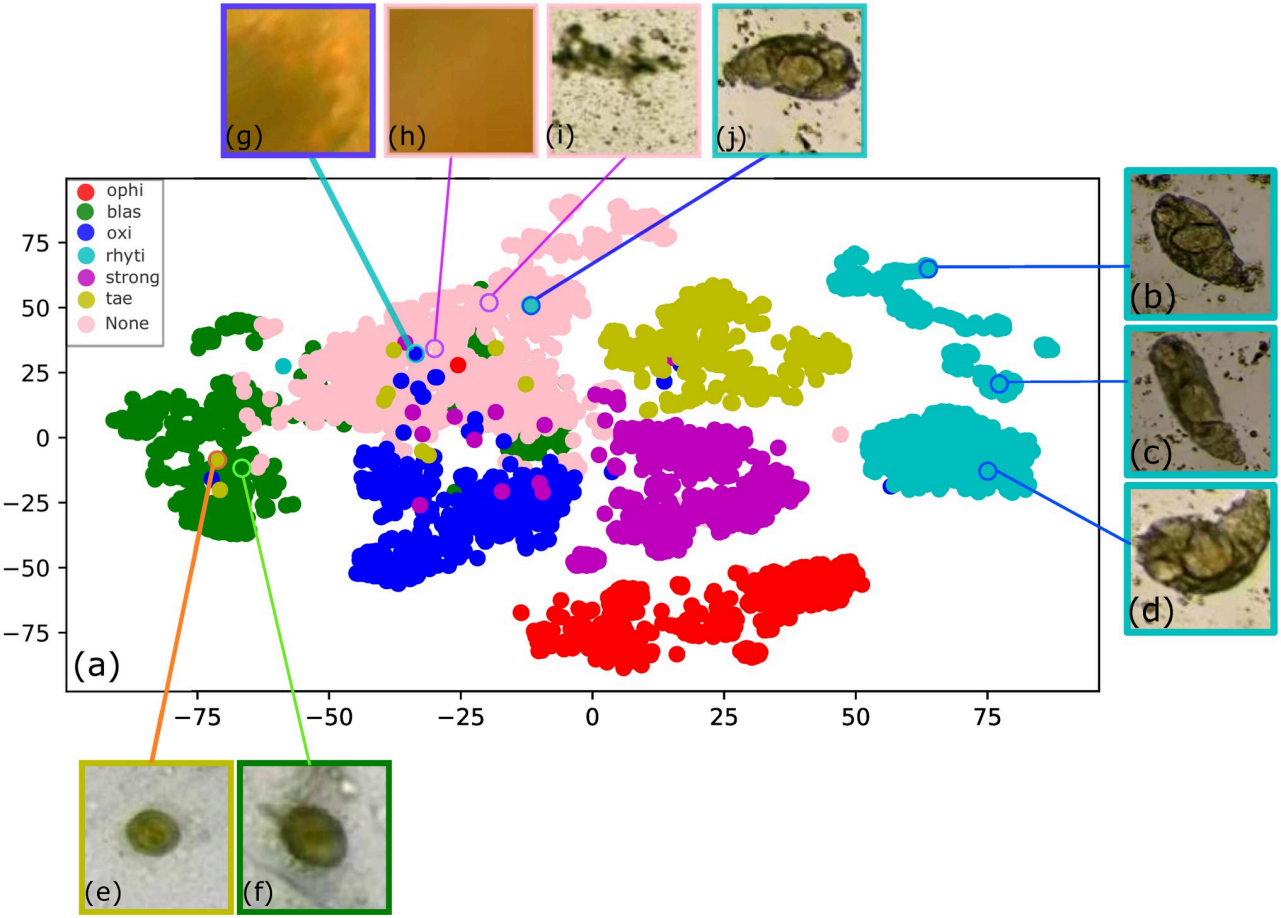
a) The T-SNE embedding shows that MobileNet is learning a meaningful representation of the image classes. On the other hand, Figs b), c), and d) show representative parasites from three different clusters of the rhyti class. Meanwhile, Figs e) and f) depict some examples of the tae and blas parasites, respectively, from overlapping regions to show their similarity. Finally, Figs g) through j) show how the neural network struggles to discriminate a parasite from background noise.

The two-dimensional embedding learned by MobileNet before the softmax layer is depicted in Fig 8. Note that the learned features form quite distinctive clusters for the six parasite classes, demonstrating that MobileNet can learn a helpful representation from the images. However, the embedding also shows how challenging is for the neural network to discriminate the absence of a parasite (i.e., the None class) due to their very similar visual characteristics with respect to some parasite instances, as shown in Fig 8g through Fig 8j.

Also, it is worth noting that there is some overlapping among classes due to similarities in morphology and color. For instance, observe in the T-SNE embedding how some blas images overlap tae images due to their visual similarities, as depicted in Fig 8e and 8f for tae and blas classes, respectively.

Also, note that the T-SNE embedding tends to form three different clusters for the rhyti class. The parasite’s natural motion can explain these three rhyti clusters during the collection stage. To exemplify this, observe the three representative rhyti examples from Fig 8b–8d taken from these three rhyti clusters. When the parasite was observed by microscope, it might be in a retracted position as shown in Fig 8b. As the parasite’s motion evolves, the parasite might take a more elongated shape as shown in Fig 8c. Finally, this parasite might also be often found in a curved shape which is its feeding position as shown in Fig 8d. Here, it’s important to note that the neural network could learn this parasite’s motion stages during training.

**Fig 9:**
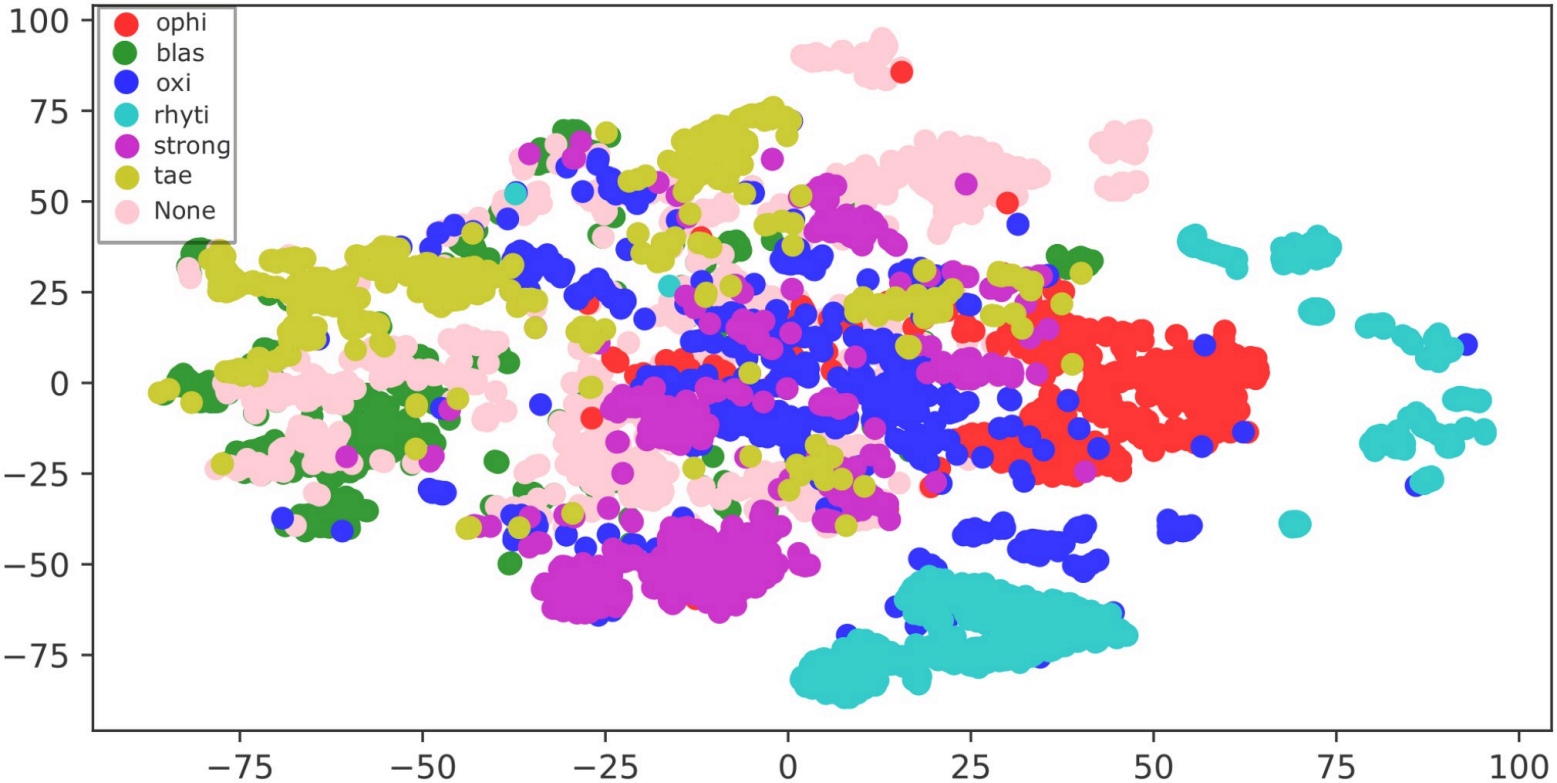
T-SNE plot of the multiclass model for the optimized custom net. Observe how the classes overlap far more when compared to MobileNet’s T-SNE.

The T-SNE embedding for the optimized custom CNN can be seen in Fig 9. Although the T-SNE plot tends to group similar parasites, note how the classes overlap far more when compared to MobileNet’s T-SNE. Concretely, classes strong, oxi, and tae are confused with each other more frequently. There is also confusion between tae and blas to a lesser degree. Moreover, the None class is often confused with the majority of the parasites, even more than the MobileNet’s T-SNE.

**Fig 10:**
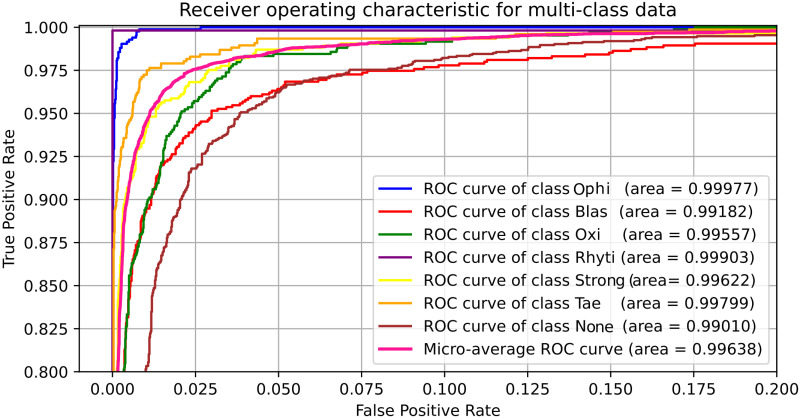
ROC plot for the MobileNet model.

**Fig 11:**
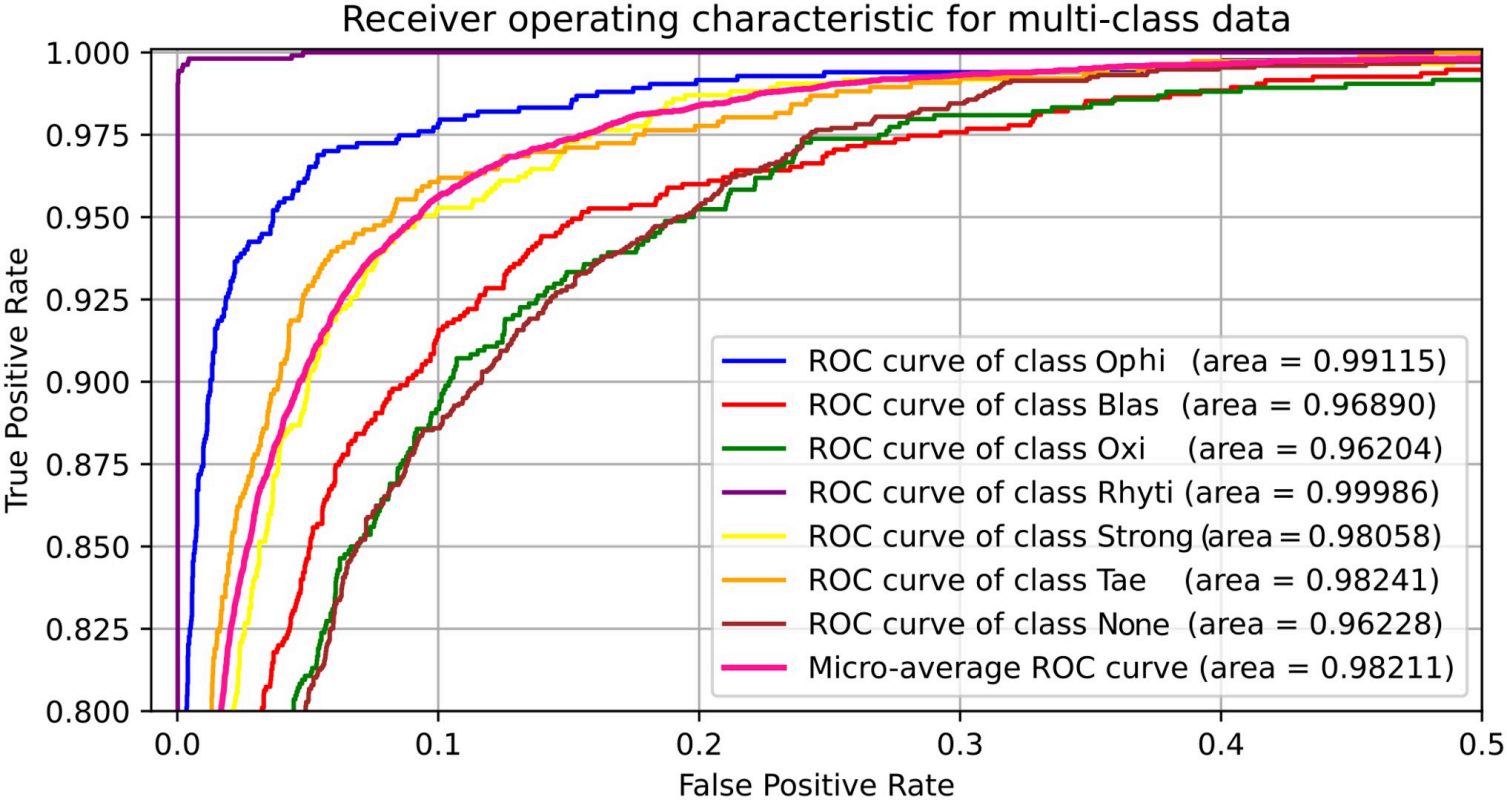
ROC plot for the optimized custom CNN.

The representation of True Positive Rate (TPR) vs. False Positive Rate (FPR) by ROC curves of each class for MobileNet, as depicted in Fig 10, shows that the lowest AUC of 99.010% is achieved for the None parasite and the highest AUC of 99.977% for the ophi parasite. Also, it shows the micro average that adds the contributions of all the classes before calculating the average accuracy, obtaining a value of 99.638%. The Fig 11 shows similar ROC annotations for the optimized custom CNN, obtaining the lowest AUC of 96.228% for the None class and the highest AUC of 99.986% for the rhyti class), and a micro average value of 98.211%.

**Fig 12:**
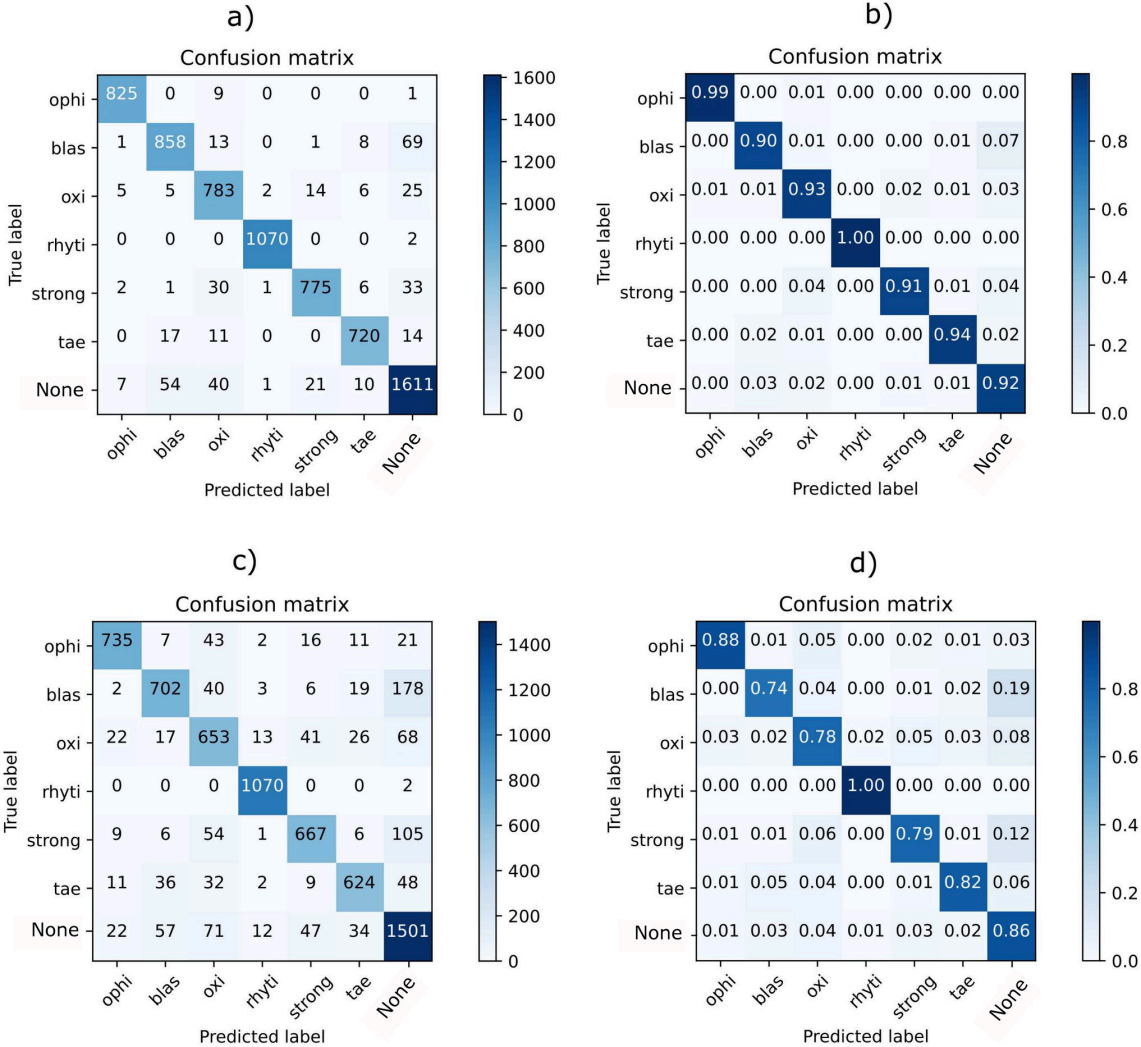
a) MobileNet’s confusion matrix b) MobileNet’s normalized confusion matrix c) Optimized custom CNN’s confusion matrix d) Optimized custom CNN’s normalized confusion matrix.

In Fig 12a and 12b, we can see the confusion matrix and the normalized confusion matrix obtained with MobileNet, respectively. Observe that for all classes the performance is above 90%. As expected from Fig 8, the confusion matrix shows how the neural network struggles more to distinguish the absence of parasites because there are background objects (i.e., debris or garbage) very similar to parasites. According to our expert veterinarian, even for specialist it is sometimes hard to discriminate the parasite from background noise but having an accuracy above 90% makes our system very suitable to perform faster diagnoses to treat captive reptile diseases. Also, a high degree of confusion is observed between the oxi class and the blas and strong classes. Some confusion is also observed between tae and blas classes. This result was expected since both classes overlap in the T-SNE embedding.

Concerning the optimized custom CNN, Fig 12c and 12d shows its confusion matrix and normalized confusion matrix, respectively. When compared to the MobileNet, the optimized custom CNN has lower accuracy across all classes. Specifically, the oxi class has a high confusion with the strong and blas classes and moderately with ophi class. Likewise, blas is often confused with strong and tae. In general, there is confusion between all classes except for the rhyti class. Moreover, the optimized custom CNN has far more trouble than the MobileNet’s approach to distinguish the absence of parasites, specially from blas, strong and oxi parasites. This result was expected from what has been seen previously in the T-SNE embedding as shown in Fig 9.

Table 4 shows the accuracy for both the transfer learning scheme with MobilNet and the optimized custom CNN (i.e., 128 filters and 6 convolutional blocks). Observe that the accuracy value obtained by MobileNet (94.26%) is better than the optimized custom CNN. We perform a t-test to confirm whether this improvement is statistically significant. The t-test confirms that the mobile network statistically outperforms the optimized custom CNN at a 0.05 significance level, according to the p-value shown in Table 4.

**Table 4:** Accuracy, standard deviation and p-values. The accuracy is the average across the 20 runs with different random data partitions. P-values are calculated using the optimized custom CNN as pivot.

Approach	Accuracy	Standard %	P-value Deviation
MobileNet	94.26	0.56	2.5e-8
Optimized custom CNN	83.96	1.63	-

**Fig 13:**
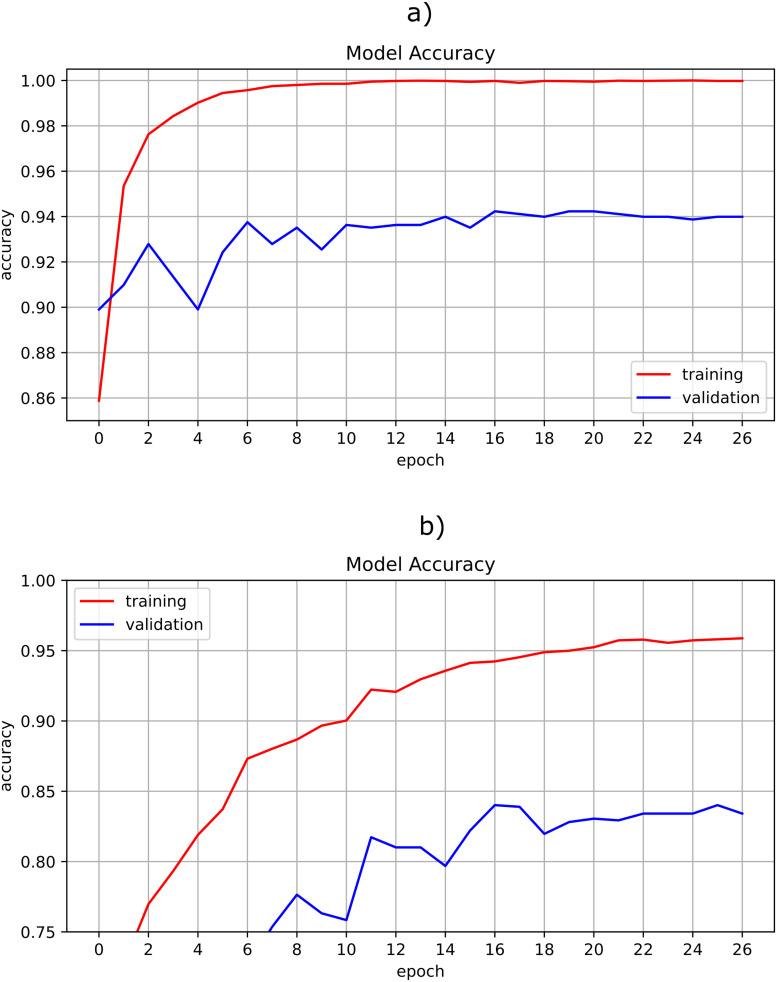
Learning curve for a) MobileNet, b) optimized custom CNN.

Fig 13a and 13b show the learning curves for the transfer learning scheme and the optimized custom CNN, respectively. With MobiletNet, the neural network achieves better performance before 10 epochs during training, while the optimized custom CNN takes more than 30 epochs. This is because MobileNet, being a pre-trained network, does not learn from scratch as the custom CNN does it.

Finally, in Table 5 we analyze the performance of our image segmentation stage. We passed the 3616 database images through our segmenter obtaining the eight largest regions that hopefully might enclose the parasite. As expected, the largest area (Ranking 1 in Table 5) is the most likely to contain the parasite, accounting for 94.94% of correctly segmented images. From the second to the eighth largest regions the contributions to the segmentation accuracy are relatively small, but in conjunction they increased the segmentation performance by 1.71% (i.e., an overall segmentation accuracy of 96.65%). Observe that if we included only the largest region, the overall segmentation error would be 5.06%. When expanding to the eight largest regions, the overall segmentation error was reduced by a third to 3.35%.

**Table 5:** Segmentation accuracy for the eight largest areas found by the image segmentation stage. Ranking 1 represents the largest area, ranking 2 represents the second largest area and so on.

Largest Area Ranking	% of Correctly Segmented
1	94.94%
2	0.39%
3	0.39%
4	0.17%
5	0.17%
6	0.28%
7	0.11%
8	0.22%
**Overall Segmentation Accuracy**	**96.65%**
**Overall Segmentation Error**	**3.35%**

### Limitations

**Fig 14:**
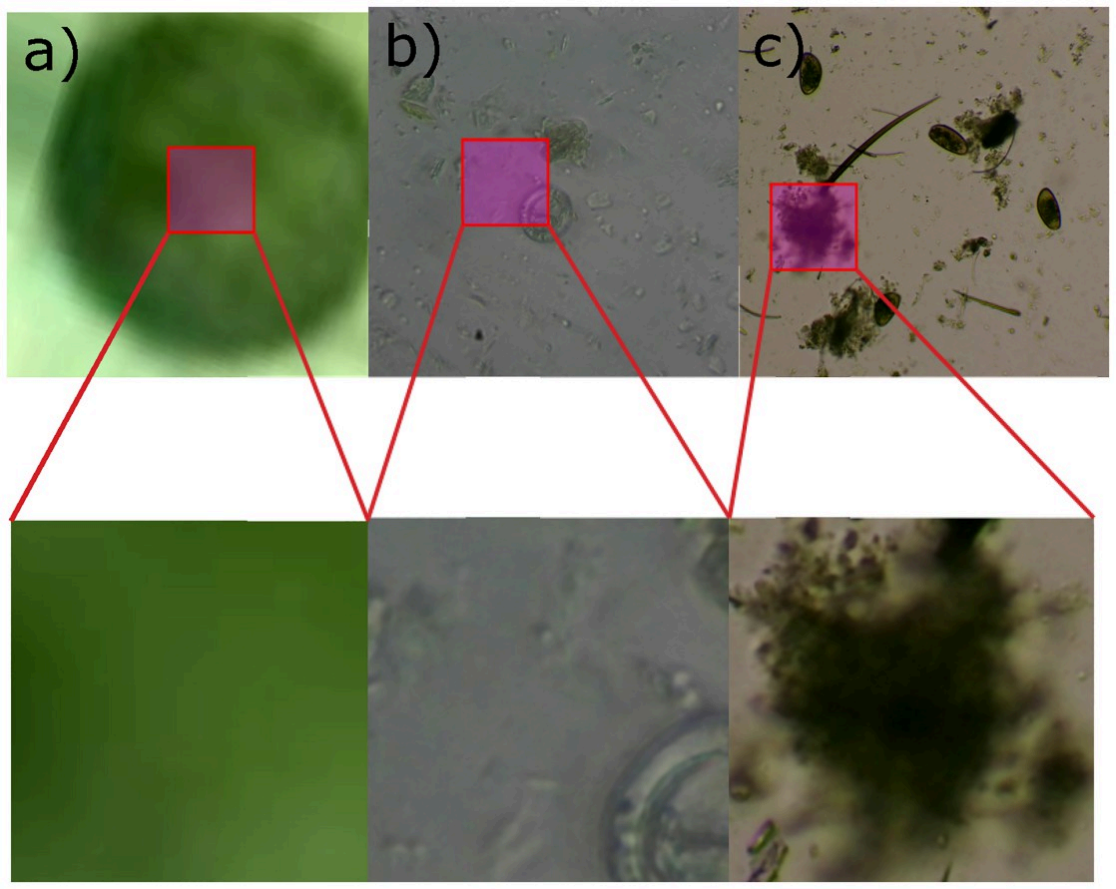
Examples of wrong segmentation.

Our approach also presents some limitations that decrease its performance due to wrong segmentation or wrong classification predictions. Some wrong segmentation examples are shown in Fig 14. Concretely, an interior region from a blas parasite was incorrectly segmented in Fig 14a. Moreover, a blas parasite was partially cropped during segmentation in Fig 14b. Finally, the parasite was completely missed in Fig 14c and instead some debris was captured by the segmentation stage.

**Fig 15:**
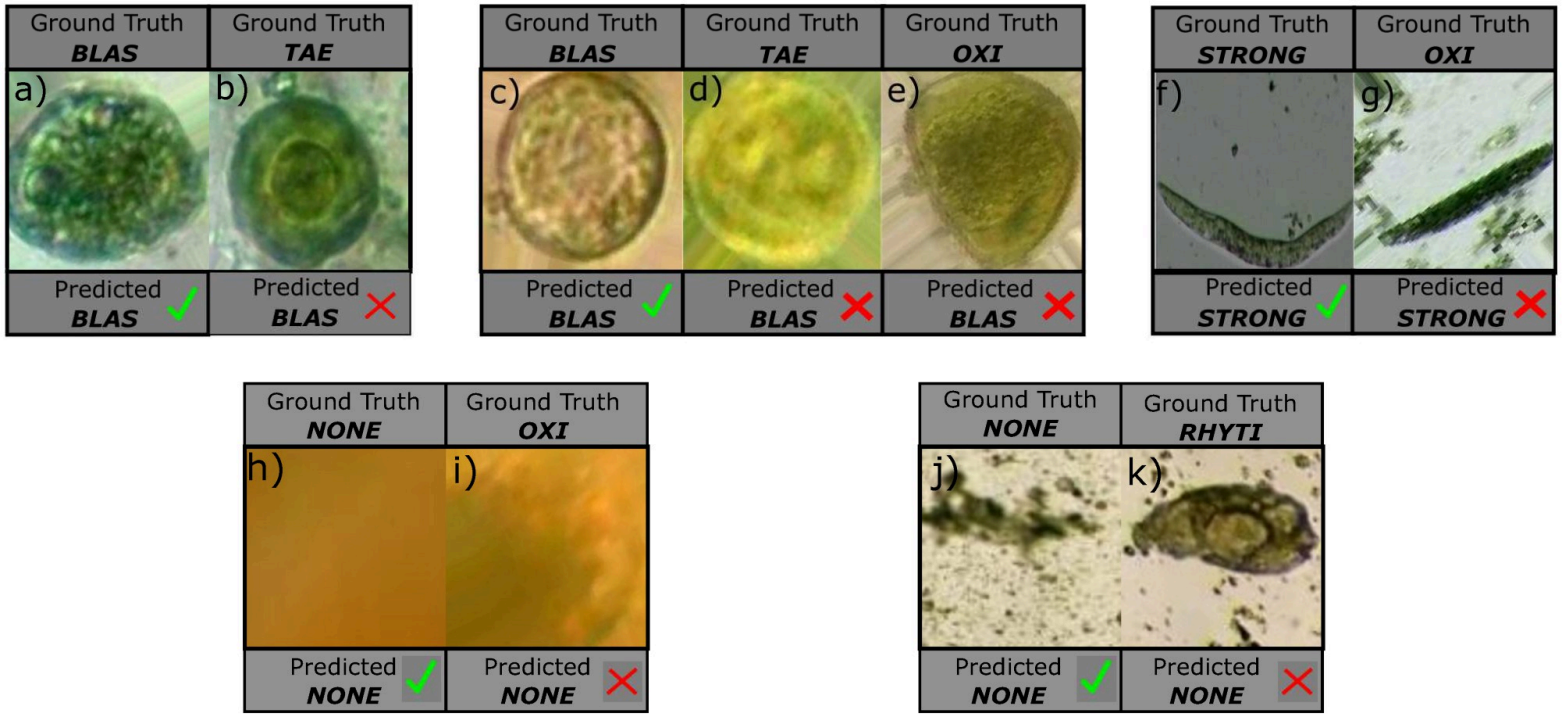
Examples of wrong predictions.

Wrong classification predictions also occurred as shown in Fig 15, where visually similar parasites are confused. For instance, our approach struggles to differentiate the granules in cytoplasm found on blas parasites, as shown in Fig 15a, which in turn may cause confusion with the tae class as shown as Fig 15b. Moreover, our neural network is sometimes not capable of recognizing the tae visual patterns such as its onchosperal membrane as shown in Fig 15d, which again leads to misclassification as blas. With respect to the oxi parasite from Fig 15e, it is incorrectly classified as blas since our method is not distinguishing the tae’s oval shape from the blas’ circular shape. In the same figure, it also seems that the similarities between the blas parasite and the tae’s embryon increase the classification error. Moreover, the oxy example from Fig 15g depicts an elongated oval shape which may be confused with the similar shape from the strong larva in movement. Finally, observe in Fig 15h that a rhyti parasite is visually similar to background noise, causing a wrong prediction. In this example, the shape and specially the color are confused with background noise because the neural network is not capable of distinguish the inner details of the rhyti parasite.

### Comparison with other intestinal parasites identification methods

Although, to the best of our knowledge, our approach is the first attempt to develop a machine learning model to specifically classify reptile intestinal parasites using CNNs from microscopic stool images, in order to explore the novelty and significance of our method, we compared it with existing advanced approaches for intestinal parasite identification in fecal samples in other species, such as mammals, as shown in Table 6. The results suggest that the proposed approach detects and identifies intestinal parasites in fecal samples with comparable accuracy.

**Table 6:** Performance comparison with other fecal parasites identification methods.

Study	Parasite Detection in	Parasites	ACC
Nagamori et al. 2020 [13]	Cats and Dogs	*Ancylostoma*	93.8
		*Toxocara*	
		*Trichuris*	
		Taeniid eggs	
Li et al. 2019 [14]	Sheep	Ascarid	92 to 96
		*Trichuris spp.*	
		Strongyle	
		*Coccidia*	
Roder et al. 2020 [15]	Humans	Helminth eggs	94
		Helminth larvae	
		Protozoan cysts	
Li et al. 2020 [16]	Humans	Hookworm eggs	92.16
		Ascarid eggs	
		Whipworm eggs	
**Proposed method**	Reptiles	*Ophionyssus natricis*	94.26
		*Blastocystis sp*	
		*Oxiurdo egg*	
		*Rhytidoides similis*	
		*Strongyloides*	
		*Taenia*	

## Conclusion

The proposed system for classifying parasitic agents was designed to obtain an adequate performance using a resource-efficient model like MobileNet. Image segmentation mechanisms were a vitally important task since most images had debris or garbage introducing excessive noise due to the nature of their acquisition. Since segmentation errors might occur, the inclusion of a *None* class for training the neural network has proven to be very suitable to avoid false positives, i.e., wrongly classifying background noise as parasite classes. The use of data augmentation through video frames for the training stage was crucial to improving network performance due to limited database size. Also, data augmentation algorithms addressed the unbalanced class problem found in this dataset. Our results show that the MobileNet outperforms a optimized custom CNN trained from scratch, demonstrating that transfer learning schemes are suitable to learn relevant features in this domain. Since obtaining labeled data of reptile parasites is an intensive and mainly manual task performed by experts veterinarians, it should be interesting to explore more advanced data augmentation techniques, such as those using the generative adversarial network (GANS) [42]. Moreover, a future study could explore new forms of segmentation (e.g., U-net segmentation [43]) that could improve overall system performance.

## Supporting information

S1 File Instructions for executing the source code. (PDF)
