# Supplementary material for: Automatic identification of intestinal parasites in reptiles using microscopic stool images and convolutional neural networks
Source: PLoS One. 2022 Aug 4;17(8):e0271529. doi: 10.1371/journal.pone.0271529 (PMC9352023; doi:10.1371/journal.pone.0271529)
Supplement: S1 File — (PDF) [file pone.0271529.s001.pdf]

Automatic identification of intestinal parasites in reptiles using microscopic stool images and convolutional neural networks

Data repository URL

Please, download this repository to your computer. The repository includes de dataset and python scripts.
[Dataset and Source Code PLOS ONE]

Getting Started

This document describes the usage of the scripts and datasets to reproduce the results from the manuscript entitled "Automatic identification of intestinal parasites in reptiles using microscopic stool images and convolutional neural networks".
The images and videos taken by the microscope are located in the directory `./dataSET1500/BASE1` (hereinafter `BASE1` dataset). The same images taken by the microscope and the frames extracted from the videos using the Sony Vegas Pro 11 video editor can be found in the directory `./dataSET1500/BASE2` (hereinafter `BASE2` dataset). This latter dataset is the one necessary to run the scripts.

For the proper execution of the scripts, please follow the instructions below:

1. The following jupyter notebooks are included, which should be located in your working directory (i.e., directory `./`):
   - `SEGMENTATION + DATA AUGMENTATION.ipynb`
   - `Reptile parasites MOBILENET notebook.ipynb`
   - `Reptile parasites CUSTOM CNN notebook.ipynb`

2. Make sure that the `BASE2` dataset is located in the following directory:
   `./dataSET1500/BASE2`

3. Make sure that within the `BASE2` folder there are three folders: a folder called `Images` with 3616 images, a folder called `Frames` with 4849 images of parasites extracted from the videos and a folder `NP` with 1744 images of background noise (no parasite class).

4. It is mandatory to install the following versions of python libraries in a conda virtual environment:
   - `Numpy 1.20.1`
   - `Tensorflow 2.3.0`
   - `Matplotlib 3.3.4`

- `OpenCV 4.0.1`
- `Pandas 1.2.4`
- `Keras 2.4.3`
- `Scikit-learn 0.24.1`
- `More-itertools 8.7.0`
- `Scipy 1.6.2`

5. The first notebook to run is the `SEGMENTATION + DATA AUGMENTATION.ipynb`. This notebook implements the segmentation and data augmentation stage. After running this notebook, the `BASE3` and `BASE4` folders and the `baseFrames.csv` and `baseImages.csv` files are generated in the directory `./dataSET1500`. The `baseFrames.csv` and `baseImages.csv` files contain the labels and names of the frames and images, respectively. The `BASE3` dataset is the result of the dataset segmentation stage from the `BASE2` images. The `BASE4` dataset is the result of the data augmentation stage from the `BASE3` images. The latter dataset is the input to the neural network training.

6. There are two jupyter notebooks that implemented the transfer learning scheme with MobileNet and the Custom CNN, respectively:
   - `Reptile parasites MOBILENET notebook.ipynb`
   - `Reptile parasites CUSTOM CNN notebook.ipynb`

   Observe that these notebooks can be run independently.
   There are several variables that can be changed depending on which test you want to perform:
   - `RSEED`: variable that defines the random seed for the k fold cross validation (both scripts).
   - `batch_size`: variable that defines the batch size (both scripts).
   - `N_Epochs`: number of epochs for the training stage (both scripts).
   - `nFilters`: number of filters (CUSTOM CNN only).
   - `nBlocks`: number of convolutional blocks (CUSTOM CNN only).

   Note 1: To obtain the best results shown in the paper, it is recommended to define these variables as follows: RSEED = 2, batch_size = 32, N_Epochs = 50, nFilters = 128, nBlocks = 6.
   Note 2: We highly recommend running the scripts on GPU hardware. Even on a GPU, you should expect the running time to be several hours.

   After running the aforementioned scripts, the variables and models are stored in the directory `./dataSET1500/Test/`
   The confusion matrices and ROC curves are displayed at the end of each script.
